# Advances in Non-Invasive Blood Pressure Monitoring

**DOI:** 10.3390/s21134273

**Published:** 2021-06-22

**Authors:** Xina Quan, Junjun Liu, Thomas Roxlo, Siddharth Siddharth, Weyland Leong, Arthur Muir, So-Min Cheong, Anoop Rao

**Affiliations:** 1 PyrAmes Inc., Cupertino, CA 95014, USA; ljj@pyrameshealth.com (J.L.); troxlo@pyrameshealth.com (T.R.); sids@pyrameshealth.com (S.S.); wleong@pyrameshealth.com (W.L.); amuir@pyrameshealth.com (A.M.); 2 Department of Geography & Atmospheric Science, University of Kansas, Lawrence, KS 66045, USA; somin@ku.edu; 3 Department of Pediatrics, Neonatology, Stanford University, Palo Alto, CA 94304, USA; anooprao@stanford.edu

**Keywords:** cNIBP, neonate, NICU, hypertension, hypotension, non-invasive blood pressure monitoring

## Abstract

This paper reviews recent advances in non-invasive blood pressure monitoring and highlights the added value of a novel algorithm-based blood pressure sensor which uses machine-learning techniques to extract blood pressure values from the shape of the pulse waveform. We report results from preliminary studies on a range of patient populations and discuss the accuracy and limitations of this capacitive-based technology and its potential application in hospitals and communities.

## 1. Introduction

Blood pressure is a vital sign that is widely used in the diagnosis and treatment of many medical conditions. Blood pressure monitoring and management is an essential part of medical care, particularly in the treatment of chronic hypertension, critical care monitoring and trauma care.

According to the American Heart Association’s (AHA) 2017 Guidelines, 90% of US adults over the age of 65 may be hypertensive, thus leading to a large segment of the population that could benefit from blood pressure monitoring [1]. Blood pressure monitoring will be a vital part of care for virtually every one of the five million plus patients admitted annually to Intensive Care Units (ICUs) in the US [2,3]. Similarly, blood pressure monitoring will be a required part of care for virtually every one of the 130 million plus patients admitted annually to emergency departments (EDs) across the US [4,5].

The most commonly used methods for blood pressure (BP) monitoring generally involve either non-invasive inflatable cuff-based oscillometric or invasive arterial line manometric measurement.

Inflatable BP cuffs take intermittent measurements, typically limited in frequency to no more than one measurement every 5 min, due to patient discomfort and the risk of skin and nerve damage. Automated systems similar to those used for ambulatory BP monitoring (ABPM) may be used but units can be expensive. Most importantly, intermittent measurements may not be sufficient for critical care where the status of a patient can change from minute to minute.

Invasive arterial lines (IALs) are able to provide continuous BP measurements and are typically used in ICU settings as they can be difficult to correctly place and maintain and require highly skilled medical staff and equipment. IALs can be difficult to use where patient transport is required, and they carry risks of complications due to their invasive nature.

BP monitoring is vital to the care of critically ill patients since episodes of both hypertension and hypotension have been linked to greater mortality and unfavorable non-mortality outcomes [6,7,8,9,10,11,12]. Even short durations of hypotension may lead to traumatic brain injuries or complications due to a lack of perfusion [7]. However, it can be difficult to obtain accurate BP measurements, particularly from children and infants born prematurely who have fragile skin, extremely small blood vessels, and very low mean blood pressure values [13,14,15,16,17].

Frequent BP measurements are also essential in emergent or trauma care. Continuous monitoring is used primarily in critical care where better BP monitoring and faster responses to correct BP excursions can lead to better outcomes [6,18,19,20]. One study showed that patients with a single episode of prehospital hypotension were 12.4 times more likely to require immediate procedures than the normotensive controls and were also more likely to require additional procedures, ICU admission, and longer hospital stays [8]. Other studies showed that the integrated depth and duration of hypotensive episodes could be correlated to mortality [9] and non-mortality outcomes [10]. BP monitoring may also have a role in non-critical patients in whom changes in BP may provide earlier assessment of increased pain or agitation [21].

Outpatient BP management is important for many medical conditions. Stroke or cardiac events can be significantly reduced through tight BP control [22]. Mortality from uncontrolled hypertension doubles for every increase of 20 mmHg in systolic BP (SBP) [23]. The CDC estimates that only 25% of the 100 M hypertensive adults in the US adequately control their BP [24] and have set an aggressive target to raise that value to 80% by 2025 [25]. Hypertensive disorders of pregnancy are among the leading causes of maternal mortality and premature births [26,27].

The annual economic burden of hypertension has been estimated as $48.6 billion per year in the US [28]. In older adults, high blood pressure increases risk of many severe health concerns, including heart disease, congestive heart failure, ischemic stroke, and cerebral hemorrhage, vascular dementia and Alzheimer’s disease [29]. This problem persists in spite of the fact that multiple classes of low cost and effective drugs for reduction of hypertension have been available for decades. It seems that further progress in hypertension control requires improved understanding on the part of care providers and improved engagement on the part of the patient [30].

Currently, the AHA recommends that healthcare professionals confirm in-office diagnoses of high blood pressure with home self-measurement and/or 24-h ambulatory blood pressure monitoring (ABPM) [1]. By giving physicians a look at blood pressure during patients’ daily lives, ABPM more accurately diagnoses hypertension and better predicts associated risks [31,32,33,34]. In older adults, patient use of ABPM has even been associated with improved cognitive function [35]. ABPM is especially useful in diagnosing “white-coat hypertension” (when a patient has elevated blood pressure when tested at a medical facility) and “masked hypertension” (when a patient has normal blood pressure in a medical clinic, but elevated outside) [31,32,34,35]. Thus, ABPM is helpful in preventing both overtreatment of hypertension, leading to falls and other accidents, as well as undertreatment [36].

### 1.1. Current Standard of Care for Blood Pressure Monitoring

#### 1.1.1. Invasive Arterial Catheters

Although cuff-based measurements are used today to guide most treatment decisions due to an abundance of literature and its ease of use, the “gold” standard for continuous BP monitoring is an invasive arterial line (IAL) [11,37,38]. This is a sensor that is inserted into the artery which directly measures changes in BP, generally after calibration with an inflatable cuff measurement. Approximately 8 million IALs are placed annually in the US [39] and in up to 2% of all births [40], but each placement carries the risk of serious complications such as infection, bleeding, clots and nerve damage [41]. IALs are generally used in ICUs due to the possibility of excessive bleeding and require maintenance to ensure patency. Costs can be high, over $750 per adult patient and over $2000 for neonates, due to the need for highly skilled medical staff and equipment [42].

While insertion of an IAL is considered essential for many critically ill patients and is generally benign for adults [12,37,38], it is more problematical for children who have smaller arteries and a greater tendency for vasospasm and must generally be sedated [11,12,15,41,43,44,45,46,47,48]. Neonates are particularly at risk due to their fragile skin [49]. Insertion of a pediatric arterial line requires the services of highly trained personnel and may require multiple attempts [12,15,43]. As many as half of pediatric arterial lines may be positioned non-optimally to such an extent that their accuracy may be insufficient to manage care [11]. Short-term and long-term complications such as obstruction of blood flow and possible tissue or organ damage from arterial thrombosis or vasoconstriction [15,41,43,44,47], infection [15,46], bleeding complications [47], and nerve damage [12] occur at a higher rate for children than adults, with younger, smaller children more likely to experience these issues [12,41,43,48]. Thrombosis has been reported in 24–66% of neonates with umbilical arterial lines in place [50]. In extreme cases, amputation may be required [51].

Despite the advantages to care management from continuous BP monitoring, the higher risk of complication leads many clinicians to avoid the use of IAL monitoring if possible, substituting intermittent cuff measurements or eschewing BP information entirely.

#### 1.1.2. Inflatable Cuff Measurements

Inflatable cuffs measure blood pressure by inflating a cuff wrapped around a limb, determining the pressure required to obstruct blood flow through the arteries in that limb, gradually reducing the pressure in the cuff and determining the pressure at which blood flow is reestablished. The first pressure at which blood flow is stopped is the systolic blood pressure (SBP) and the value at which blood flow resumes is the diastolic blood pressure (DBP).

Cuff measurements can be time consuming if taken manually, and placement can be critical to the accuracy of the results. Frequent use of cuff devices is uncomfortable and carries risk of complications such as damage to the skin (e.g., petechiae, acute dermis capillary rupture, or skin necrosis) or peripheral nerves (e.g., compressive neuropathy, crush syndrome, or nerve ischemia) [52,53,54]. Cuffs are also less accurate at children’s lower BP values [55]. Most importantly, intermittent measurements may not be sufficient for critical care where the status of a patient can change within minutes.

There is strong demand for a safer, non-invasive alternative which could be used for the majority of critically ill patients who do not require frequent blood gas sampling. More frequent BP monitoring could become ubiquitous during the 0.5 million pediatric surgeries in the US each year [56] and ultimately could become routine in ICUs and step-down wards, particularly if the monitoring device is unobtrusive and does not interfere with the patient’s sleep or activity. This could impact the care for 400 thousand critically ill neonatal patients (10% [57] of 4 million live births [58]) and 3.5 million pediatric admissions in the US each year [56].

In the long-term, use of a comfortable, non-stressful, and easy-to-use BP monitor could spread to other applications as well. It potentially could be used to screen for some congenital heart defects such as patent ductus arteriosus, coarctation of the aorta, and aortic valve stenosis using BP and pulse waveform measurements at different body locations [59,60,61,62,63,64,65,66,67]. With a low price point, it could be used for routine BP measurements during office visits. It could also be used to assist in the diagnosis and care management of hypertensive patients, both children and adults, and the use of ambulatory BP monitoring for children is now encouraged by the American Academy of Pediatrics [68].

Home BP measurements improve BP control. Since use of arterial lines is limited to critical care facilities, multiple home BP measurements and/or 24-h ambulatory BP monitoring (ABPM) using automated brachial cuffs, are used to provide an indication of BP variation during patients’ daily lives. The AHA recommends using ABPM to confirm in-office diagnosis of hypertension, and it is especially useful in diagnosing “white-coat hypertension” (when a patient has high BP when tested by a medical professional) and “masked hypertension” (when a patient has normal BP in a medical clinic, but elevated BP outside the clinic) [1]. Thus, home BP measurement, particularly ABPM, is helpful in preventing both overtreatment of hypertension, leading to falls and other accidents, as well as undertreatment. It can also improve patient care through titrated treatment schedules for more precise BP control and has also been shown to be a useful tool in the diagnosis and prediction of gestational hypertension (GH) [69].

BP monitoring is difficult to implement outside of a hospital; despite the utility of ABPM, it is not widely used. It is estimated that fewer than 1% of Medicare patients are prescribed ABPM to manage their BP [70]. Several factors prevent widespread use by providers and patients [31]. The primary barrier is patient compliance and a reluctance or inability to obtain measurements with sufficient frequency. Brachial cuff devices are cumbersome and can be uncomfortable, leading to incorrect or insufficient usage [71,72,73]. Measurements with ABPMs can be variable, inaccurate and inconvenient for patients. They also interfere with patients’ sleep and activities [31,32,35,73,74]. The devices are expensive–up to $3000 per unit–and may not be covered by health insurance [31,71]. Moreover, most patients cannot tolerate using an ABPM for more than 24 h and long-term usage can lead to tissue damage. For these reasons, traditional ABPM devices are less than ideal for long-term monitoring, such as monitoring women for hypertension throughout their pregnancy.

There is a clear need for a convenient, continuous, and cost-effective device that can enable widespread BP monitoring with minimal risk, training and effort. PyrAmes has developed a comfortable and easy-to-use wearable device for BP monitoring and management over long periods of time. It uses capacitive sensors to capture pulse waveform data which can provide more detailed information about the health of the cardiovascular system. It has been used successfully on neonates.

### 1.2. Current Alternatives in Blood Pressure Measurement

There have been many attempts to develop more convenient alternatives to address the limitations of current BP measurement techniques (Table 1). We separate them into cuff-based and cuffless non-invasive devices.

#### 1.2.1. Cuff-Based Devices

The most successful alternatives for monitoring adults are volume clamp devices worn on the fingers and calibrated with a standard inflatable cuff measurement [75,76,77]. These volume-clamp devices partially inflate the finger cuffs using a photoplethysmographic (PPG) absorbance as the metric for arterial expansion. The pressure required to minimize changes in arterial expansion and keep the blood volume constant during each pulse enables reconstruction of brachial BP after calibrating against a brachial oscillometric NIBP value. These devices may include proprietary algorithms to autocorrect vascular tone changes related to vasodilation, vasoconstriction, and use of vasoactive drugs.

Volume-clamp devices currently on the market include the Nexfin/ClearSight (Edwards Lifesciences, Irvine, CA, USA) [78], CNAP (CNSystems, Graz, Austria) [79], LiDCO Blood Pressure Module (LiDCO, London, England) and Finapres/Finometer (Finapres Medical Systems, Enschede, The Netherlands) systems [76]. These devices are bulky, uncomfortable, inconvenient, expensive, and not widely used. The Caretaker device (Caretaker Medical NA, Charlottesville, VA, USA) [80] is wearable but interferes with the use of the hand and is expensive ($5 K).

Wrist-worn inflatable cuffs (Heartguide (OMRON Healthcare, Kyoto, Japan) [81], H2-BP (Charmcare, Seoul, Korea) [82]) have the form-factor of a watch and do not interfere with the use of the hand. However, they provide only intermittent BP data. Moreover, frequent use of cuff devices can cause skin or nerve damage [52,53,54]. Importantly, like conventional cuff-based methods, these devices lose accuracy when mean arterial pressure (MAP) is <30 mmHg [83], a level very common in sick neonates. Some of these devices meet guidelines for accuracy cited by the US Food and Drug Administration (FDA) and the European Union (EU), i.e., a mean average error (MAE) or mean difference $\left(\bar{X}n\right)$ <±5 mmHg and standard deviation (sd) <8 mmHg [84,85].

#### 1.2.2. Cuffless Devices

Cuffless devices have used mechanical and optical sensors to determine pulse transit time (PTT), pulse wave velocity (PWV), the pulse contour, or the acceleration pulse to extract BP values [86,87]. Some mechanical “cuffless” devices use arterial applanation tonometry to clamp an artery between a transducer and bone. Arterial applanation tonometry places a pressure transducer at a pulse point and gently compresses the underlying artery. The tonometer uses algorithms to estimate the arterial wall tension and quantify the arterial pulse. They generally require initialization or calibration with a cuff blood pressure measurement. Examples of this are the T-Line device (Tensys Medical, San Diego, CA, USA) [88,89] and the BPro (HealthStats, Singapore) which have received FDA clearance [90].

Smartphones use PPG, electrocardiographic signals, and acceleration sensors to measure BP [91]. The user can press a finger against the device attached to the smartphone and measure blood volume variations [92] although there are reservations about the accuracy of these applications [87]. The Health Monitor app (Samsung, Seoul, South Korea) tracks heart rate and blood pressure using pulse wave analysis in conjunction with their PPG heart rate monitors on their smartwatch. It measures the blood pressure change after calibration with a cuff measurement to determine blood pressure. The Samsung app received a European Union approved Conformity Marking (CE)-marking in December 2020 [93].

PPG-based devices illuminate the skin like a pulse oximeter and measure blood volume below the skin based on changes in absorbance. Unfortunately, PPG is prone to interference from ambient light, skin pigmentation, and hair. In addition, PPG requires significant skin pressure to reduce ambient light interference which can damage the fragile skin of neonates and elderly patients. Unlike IAL waveforms, PPG pulse waveforms generally lack detailed morphologic features which are critical in inferring BP using machine-learning methods.

Despite these downsides, there have been some successes with PPG. In a study of 86 participants that included 40% with high skin pigmentation and 20% with denser hair on the forearm, the Aktiia bracelet (Aktiia SA, Neuchâtel, Switzerland) was stated to meet the accuracy and precision required for ISO 81060-2:2013 for periods as long as a month [94]. In a study of 1075 subjects with both normal and high blood pressure levels, Biobeat (Biobeat, HaMerkaz, Israel) proved accurate and reliable in alignment with the same standard [95]. The U.S. FDA has granted a 510 K clearance for its patch and watch.

Another approach is to correlate BP to the PWV-the speed of a pressure pulse propagating along the arterial wall calculated from the PTT (the time between two pulse waves propagating during the same cardiac cycle from two separate arterial sites). Because of its simplicity, it is gaining popularity for tracking BP. Companies in this space (e.g., Vital Insite, Palo Alto, CA, USA, Quanttus, Mountain View, CA, USA, Scanadu, Sunnyvale, CA, USA, Blumio, San Francisco, CA, USA) have not yet found widespread commercial success. Many of these devices have struggled to accurately coordinate multiple sensors and require a large amount of training data to describe the wide variations of vasculature and demographics of the general populace.

Sensifree (Cupertino, CA, USA) is known to use Non-invasive Electromagnetic Sensing Technology (NEST), with results significantly more similar to arterial line data than a PPG sensor (*p* < 0.0001) [96]. They have algorithms to estimate and track systolic and diastolic pressure values from the mean arterial pressure and pulse morphology.

Similar to PyrAmes, Vena Vitals (Irvine, CA, USA) uses capacitive sensing to capture pulse waveform signals which are then processed by machine learning to derive BP values. Vena Vitals’ capacitive structures utilize wrinkled electrodes deposited on elastomeric substrates which historically have been difficult to manufacture in volume.

## 2. Materials and Methods

### 2.1. PyrAmes’ Solution

**Figure 1:**
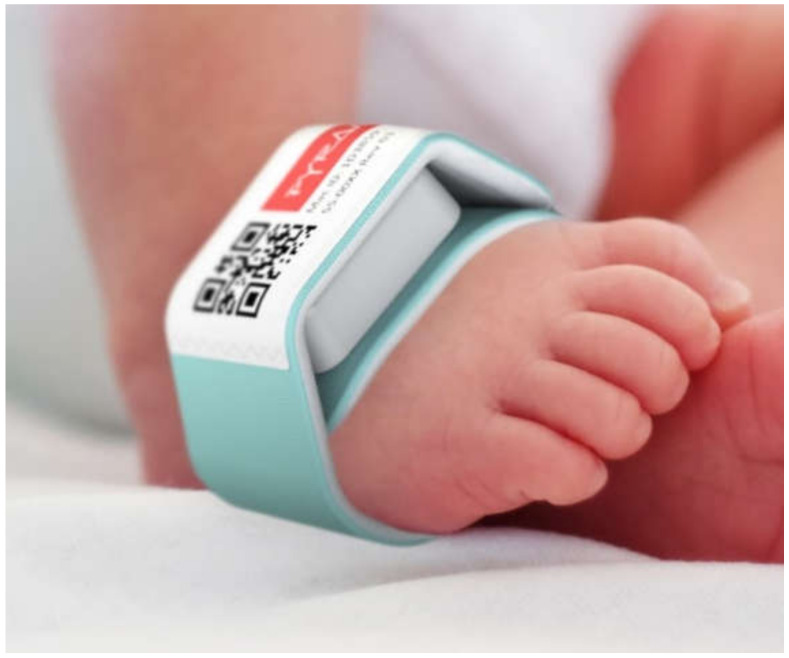
Boppli^TM^ device to monitor the blood pressure of infants in critical care.

PyrAmes uses capacitive proximity sensing technology exclusively licensed from Stanford University to collect pulse waveform data which is used to derive continuous blood pressure measurements. The system consists of an array of sensors integrated into a soft foam band which is worn on the wrist or foot (Figure 1) which sends data wirelessly to a mobile device where continuous blood pressure values are displayed in real time. The sensor has been designed to be compatible with standard roll-to-roll processes for high volume manufacturing.

### 2.2. How It Works

**Figure 2:**
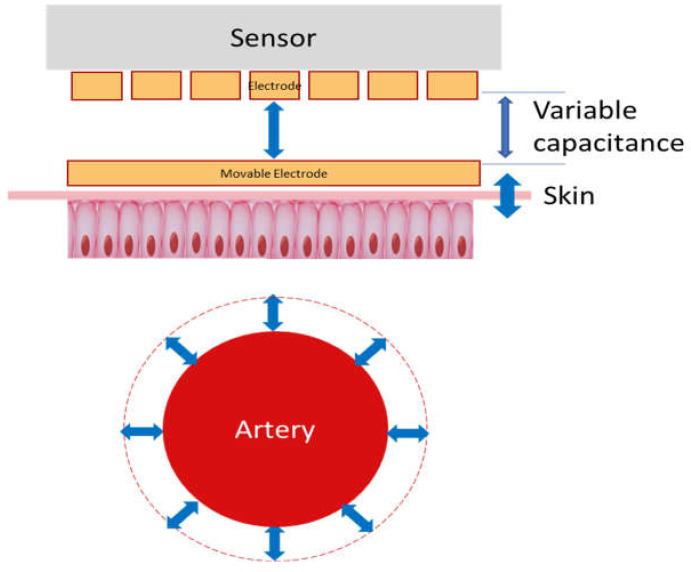
Schematic of operating principle of PyrAmes’ capacitive sensor technology.

The PyrAmes device is based on a very sensitive relative displacement sensor that sits with light contact against the skin over a palpable pulse point. When the heart beats, it pushes a pulse of blood through the arteries which locally expand and contract as the pressure wavefront caused by this pulse travels through them. This causes the surface of the skin to be displaced slightly which, in turn, results in motion of a movable surface within the PyrAmes sensor (Figure 2).

**Figure 3:**
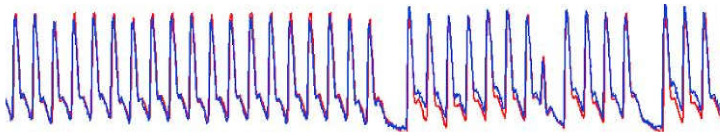
Comparison of normalized invasive arterial line data (red) taken simultaneously with normalized PyrAmes sensor data (blue).

The change in distance between the movable surface and a fixed electrode in the PyrAmes sensor is measured as a change in capacitance. The capacitance changes with time in a manner which correlates to the blood pressure changes or pulse waveforms that are measured with an IAL (Figure 3). The capacitance signal from each array element is digitized with a capacitance-to-digital converter for wireless transmission to an Android mobile device via the Bluetooth Low Energy protocol.

An array of four independent sensing elements spanning an 8 × 15 mm^2^ area is used to ensure that it is easy to locate the device over a pulse point. The firmware on the electronics can determine the signal-to-noise from each element of the array and use this information to transmit only the best signal to the Android device to extend battery life.

An application on the Android device receives the sensor data and uses a Butterworth bandpass filter to remove noise and artifacts from the data. The cleaned data is evaluated for signal quality (QA score) with a convolutional neural network (CNN) that was trained on waveform data which had been visually graded against a rubric of quality values reflecting the similarity of the sensor signal to IAL data taken simultaneously.

Data with QA scores above a set threshold value are sent to another CNN trained on a combination of normalized arterial line data and sensor data to extract blood pressure values from the shape of the pulse waveform data. Data with QA scores below the threshold are discarded. Normalized waveform data are used to ensure that the model is trained from the shape and features of the waveform which makes the sensor less affected by environmental effects such as placement pressure, sensor positioning, skin temperature, and skin hair density. Since it is not optically based, it is insensitive to ambient light and skin color. However, like many other wearable devices, it is highly sensitive to motion, and motion-affected data is currently excluded through use of the QA score. Ultimately it is expected that some types of motion artifacts will be mitigated through signal processing and the use of reference sensors. Models for different use cases may require anchoring with demographic values such as age and weight, or with a recent cuff measurement.

The premise that blood pressure can be derived from pulse waveform data is well supported in the literature. Much effort has focused on identifying features in the waveform shape that can be correlated to specific blood pressure values and other hemodynamic parameters. For example, Baruch et al. correlated the ratio of the heights of the primary and secondary peaks seen in the waveforms for many individuals, particularly adult males, to the central systolic pressure [97]. They also correlated the time between the primary and tertiary peaks in milliseconds with diastolic pressure. Munir [98] and Donley [99] report similar analyses using different features of pulse waveforms. Secondary and tertiary peaks in the pulse waveform arise from reflections of the pressure wave when the pulse wavefront experiences a change in flow, such as at a branch point in the arteries. The timing and shape of these features are related to the geometry and mechanical properties of the arteries [100]. Stiffer arteries can cause more pronounced secondary peaks that appear closer in time to the primary peak, and thus different correlations may be needed to address the data for patients of different ages, medical conditions or lifestyles.

Clinical data for these studies were collected using protocols approved by the Institutional Review Boards at Stanford University and Aspire IRB. Details of the studies are provided below.

## 3. Results and Feasibility Studies

### 3.1. Proof of Concept

To demonstrate the feasibility of the PyrAmes sensor, an early prototype was used to collect data from a male subject, >89 years old, who exhibited a wide variation of blood pressure on a daily basis. Brachial blood pressure measurements were taken while the subject was seated with both feet flat on the ground with a Series 10 Model BP786 oscillatory cuff (OMRON Healthcare, Kyoto, Japan) or Model ABPM50 ambulatory blood pressure monitor (Contec, Hebei, China) at varying intervals over a period of 7 months. There were 1 to 3 sessions per day for a total of 412 blood pressure readings. With Omron measurements, three cuff measurements were taken 1 min apart on each arm at each session. With the Contec ABPM, measurements were taken every 15 min for up to 8 h each session. Sensor data was collected with an array of 4 sensors from the opposite arm at the radial pulse point throughout the session. An array of sensors is used to ensure that at least one sensor is located near the palpable pulse point to facilitate placement of the device. It was found that using multiple smaller sensors provided more detailed pulse waveform shapes than a single large sensor with the same areal coverage. The sensor array and electronics were held in place with a knitted elastic band. The data were streamed using Bluetooth 2.0 protocol to a Windows tablet and processed with a Labview executable application.

**Figure 4:**
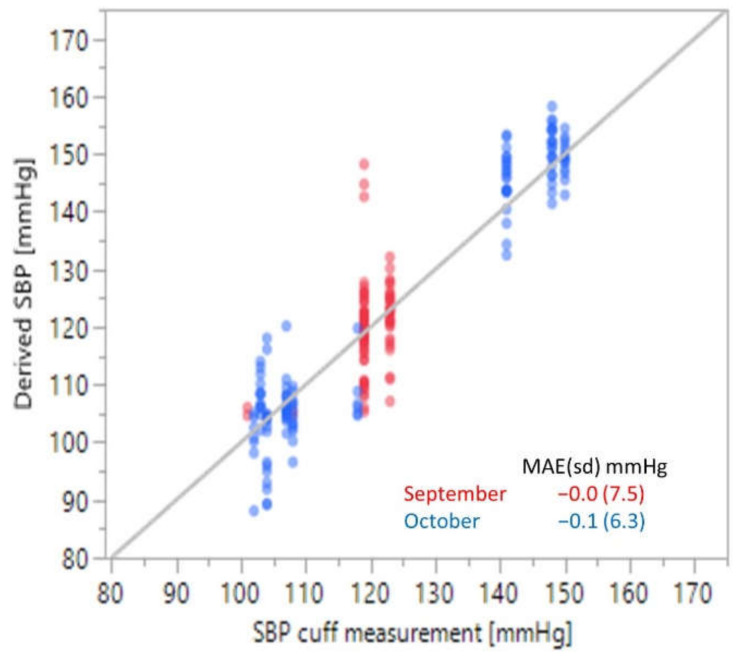
Proof-of-concept results for a single person convolutional neural network algorithm trained on cuff data collected from March through May and then tested in September and October.

For analysis, the sensor data was processed post-collection with a 0.1 to 20 Hz 1st order Butterworth filter. The data were then divided into one-minute windows bracketing the time of each cuff measurement. Since there were 4 sensors in each array, there were four data windows for each cuff measurement which were tied to the same reference SBP value. 1162 data windows were randomly chosen from the data collected during the period from March through May and used to train a CNN to extract SBP values from the normalized sensor data. The remaining 255 data windows from the two-month period were used to test the model. 230 additional data windows taken in September and October were used to validate the CNN algorithm (BP-model-1) for accuracy with the results shown in Figure 4. 

The mean average error (standard deviation) was −0.0 (7.5) mmHg in September and −0.1 (6.3) mmHg in October, well below the FDA guidelines of < ±5 (8) mmHg [84]. This suggests that the approach of using the pulse waveform data to derive blood pressure values is feasible and somewhat robust over time, potentially without additional calibration.

### 3.2. Ambulatory Adults

**Figure 5:**
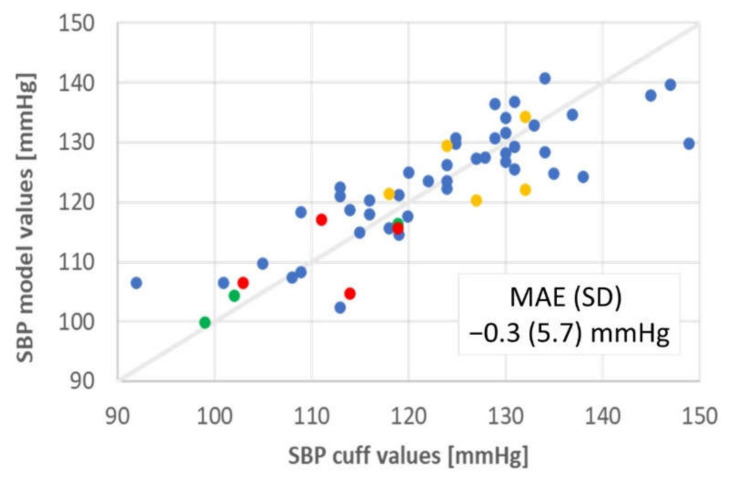
Results from convolutional neural network model trained with cuff data from Study 1 and tested with an ambulatory blood pressure monitor for 4 other ambulatory subjects (SBP values color coded by individual).

The concept was explored further in a collaboration with Drs. Vivek Bhalla and Tara Chang of the Stanford Hypertension Clinic and Dr. Sandra Tsai of the Stanford Cardiology Clinic (Study 1 in Table 2). Up to six cuff measurements were taken over the space of 10 min for each of 100 ambulatory individuals with an Omron Series 10 Model BP786 oscillatory cuff, three on each arm at the brachial position separated by 1-min intervals. PyrAmes sensor data was taken simultaneously on the opposite arm from the cuff using the same prototype as in the proof-of-concept demonstration. Subjects were seated with both arms elevated on the arm rests of the chair and with both feet flat on the ground. Usable sensor data was obtained from all 100 subjects although data was only obtained from a single arm for two individuals. Usability of the data was determined through a quality metric output by a CNN algorithm (QA-model-1). This model was trained on quality scores assigned upon visual inspection of 4-s samples of waveform data using a rubric that considered signal quality as well as similarity to typical pulse waveform shapes with quality scores ranging from 0 to 5. The one-minute interval bracketing each cuff measurement was divided into overlapping 4-s segments, each normalized using the minimum and maximum values of the waveform data in that segment. Segments with a quality above 2.5 were processed with a Butterworth filter (0.1 to 20 Hz, order 1), and used to train a second CNN (BP-model-2) to extract SBP from sensor data. The CNN algorithm was then tested without additional calibration with data from 4 adults where periodic cuff measurements were taken with a Contec ABPM50 for up to 24 h (Figure 5). The test group were all healthy individuals aged 48–68 years old and included three women and one man. The results meet the FDA guidelines for accuracy when the quality score of the sensor data is >4. However, less than 30% of the data met this criterion, particularly when the subject was moving.

### 3.3. Critically Ill Patients

While the results from Study 1 were promising, it was clear that more data would be required to train robust algorithms which can meet the FDA guidelines for accuracy for a broad population of individuals. To that end, studies were initiated with critically ill patients who had IALs in place as part of their standard of care in collaboration with Drs. Anita Honkanen, Chandra Ramamoorthy, Archana Verma, and Alexandria Joseph (Study 2a in Table 2) and Drs. William Rhine and Anoop Rao (Study 2b in Table 2), all staff members of the Stanford University Medical Center (SUMC).

In Study 2a, 120 patients ranging from 4 to 87 years old with IALs already in place were recruited from the ICU, Pediatric ICU (PICU), and Cardiovascular ICU (CVICU) at SUMC. The IAL and the PyrAmes sensor were generally located contralaterally at the radial position. A few patients had the IAL at the brachial position. Some pediatric patients had the PyrAmes sensor placed at the *dorsalis pedis* pulse point when a radial location was unavailable. On average, about 6 h of data were collected per patient from each of 4 sensors in an array of sensors spanning an area of 6 × 10 mm^2^.

In Study 2b, 16 patients ranging from 1 to 8 days old with umbilical IALs already in place were recruited from the Stanford Neonatal ICU (NICU) and CVICU. Patients had gestational ages at birth of 25–40 weeks. They weighed 0.7–3.6 kg. The ratio of male to female patients was 11:5. Mean blood pressures and ranges were 54 (25–89), 66 (34–107), and 44 (17–81) mmHg for MAP, SBP, and DBP, respectively.

The PyrAmes sensor was placed at the radial (N = 13) and/or *dorsalis pedis* pulse point (N = 5). In some cases, more than one sensor location was used. Data collection ceased when the IAL was removed as part of standard care. On average, about 10 h of data were collected per patient from each of 4 sensors in an array of sensors spanning an area of 15 × 7 mm^2^. The sensor and electronics used in Study 2b were upgraded from Study 2a to improve the sensitivity of the device and to be more appropriately sized for infants.

Pulse waveform data from the IAL and PyrAmes sensor were processed with a Butterworth filter (0.1–20 Hz, order 2). The PyrAmes sensor data from Study 2a was upsampled from 67 Hz to 125 Hz to match the sampling frequency of IAL waveform data. Both IAL and sensor data in Study 2b were collected at a 125 Hz sampling rate.

The data were parsed into 4-sec windows centered around each peak and graded for quality with a CNN (QA-model-2). The training set for QA-model-2 consisted of 10,000 4-sec windows visually labeled for quality by 10 people against a rubric which compared the sensor data against the IAL data taken simultaneously. The sensor data were synchronized with IAL data taken simultaneously by determining a global lag time between the two time series through correlation of the highest quality windows of sensor data across the entire IAL time series. A local adjustment to the global lag time was then determined for each individual window to correct for missing data or other irregularities in data transmission.

The sensor windows were labeled with SBP, DBP, and MAP ground truth values derived from the corresponding IAL windows, as well as patient age and weight, and sensor and IAL QA scores. A random sampling of 5000 waveforms with a sensor QA score above 2.5 from each individual was used to train a CNN (BP-model-3) to extract blood pressure values from the sensor data. The training set was augmented with historical pediatric IAL data from SUMC (*n* = 899) and neonatal IAL data, courtesy of Children’s National Hospital (*n* = 40), which was processed with the same filters and graded with QA-model-2. Normalized IAL data with a QA score above 4 was used in the training set. The combined clinical and historical dataset was divided into 1-vs.-all cross validation sets to maximize the utility of the restricted amount of clinical data.

Early work indicated that our training set was insufficient to derive BP values for the entire age range of Study 2a that would meet the FDA guidelines for accuracy. While mean average errors were generally between −3 and +3 mmHg, standard deviations were routinely above 10 to 12 mmHg. Significantly more training data and more complex models would be needed to reduce the spread in the results. Thus BP-model-3 in Study 2b, a CNN based on TensorFlow [101], focused on neonatal data since this was a more homogeneous population for which we had comparatively more training data. This is also a more urgent unmet need since BP monitoring with IALs for this group is more difficult and poses greater risk due to the small size of neonatal arteries [12]. It is also difficult to obtain cuff measurements on a frequent basis since neonates are prone to pressure ulcers from the pressure of medical devices [102], and best practice is to avoid leaving cuffs in place for extended periods of time [103].

In Study 2b, blood pressure values were determined with BP-model-3 from the pulse waveform data collected by the PyrAmes sensor. It was loosely anchored with initial BP values and patient age and weight. Table 3 shows how this model clearly meets the FDA guidelines for accuracy.

**Figure 6:**
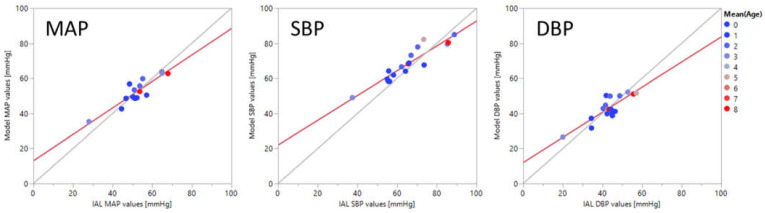
Plots of convolutional neural network model outputs vs. invasive arterial line ground truth values of mean arterial pressure (MAP), systolic (SBP), and diastolic (DBP) blood pressure values averaged over each individual. The points are color coded for the patients’ age in days.

In Figure 6, mean average values of MAP, SBP, and DBP extracted with BP-model-3 are compared against corresponding blood pressure values measured simultaneously with an arterial catheter. Each point represents the average value for all the points taken for an individual. The color of the point indicates the age of the patient in days. The derived BP values are well correlated with the ground truth values, with correlation coefficients of 0.91, 0.93, and 0.85 for MAP, SBP, and DBP respectively.

**Figure 7:**
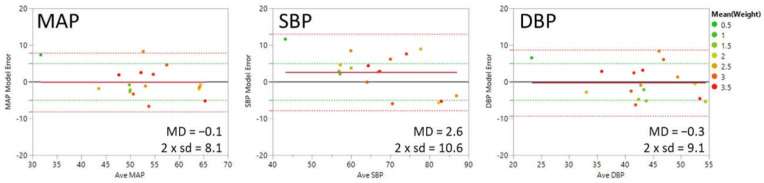
Bland-Altman plots of BP-model-3 results for mean arterial (MAP), systolic (SBP), and diastolic (DBP) blood pressure values in mmHg averaged over each individual. The *x*-axis is the average of the model and reference blood pressure values. The *y*-axis is the difference between the model and reference values. The points are color coded for the patients’ weight in kilograms. The red solid line indicates the overall error of the model (Mean Difference (MD)). The dotted red lines indicate 1.96 × the standard deviation (sd). The green dotted lines indicate the MD limits for accuracy of ±5 mmHg per guidelines from the US Food and Drug Administration.

Figure 7 shows Bland-Altman plots for MAP, SBP, and DBP with the average of model and ground truth values for each individual on the *x*-axis and the average difference between model and ground truth values for each individual on the *y*-axis (model error or mean difference (MD)). The points are color-coded by the weight of the patient in kilograms. The solid red line shows the mean average error or bias. The green dotted lines indicate the FDA guidelines for the limit of acceptable error for the population of ±5 mmHg. The red dotted lines indicate the 95% confidence interval (1.96 × the standard deviation of the error in the measurement). The mean absolute error is 4.1, 5.5, and 4.7 mmHg for MAP, SBP, and DBP, respectively.

**Figure 8:**
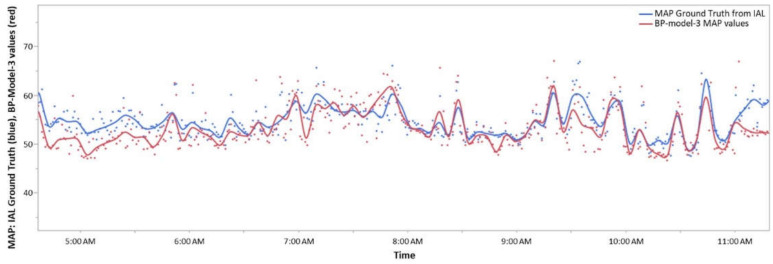
Comparison of invasive arterial line (blue) and BP-model-3 (red) mean arterial pressure (MAP) values as a function of time. Each data point is averaged over a one-minute interval. The curves are a spline fit with lambda = 1 × 10^−6^.

Figure 8 demonstrates how derived BP values (red curve) can track short term changes measured by the IAL (blue curve) over time. Data were collected for about six hours from an 8-day old infant boy (39 week 1 day gestational age at birth, 2.68 kg) with an umbilical arterial catheter in place. The Boppli sensor was placed on the left radial position. Both IAL and BP-model-3 values were averaged over 60 s intervals for this analysis. The curves are spline fits to the individual values with lambda = 1 × 10^−6^. Results for SBP and DBP exhibit similar correspondence to IAL data.

## 4. Discussion

Continuous blood pressure monitoring is essential to the diagnosis and treatment of many medical conditions but the current standards of care, invasive arterial lines and inflatable cuffs, both have serious drawbacks which impede their use in many situations. There have been many attempts to develop non-invasive approaches which address the issues faced by the current standard of care which have not yet received widespread adoption due to cost or other considerations. We introduce in this article another non-invasive approach utilizing capacitive sensing technology which shows promise in addressing some of the issues of the current alternatives. High sensitivity and a soft, smooth form-factor enables use with the fragile skin of newborns and elderly patients, two vulnerable patient populations where use of either invasive arterial lines or cuffs can be problematic. User feedback from usability studies indicates that the PyrAmes device is comfortable and easy to use, addressing issues that lead to a lack of compliance in many self-monitoring situations. Non-invasive ease of use makes the technology attractive for emergent, trauma, and pre-hospital care.

In feasibility studies, we have demonstrated that capacitive sensing technology can collect pulse waveform data that can be used in conjunction with algorithms trained with machine learning to determine accurate blood pressure values which can be stable over long time periods without additional calibration. However, for models covering a wide population range, it appears that it will be difficult to obtain sufficient training data solely with cuff measurements, and we have found it necessary to augment our training sets with clinical data where sensor and arterial line data are taken simultaneously.

The bulk of this study focused on a somewhat homogeneous population of neonates under 8 days old with an umbilical arterial catheter in place. The training set was further augmented with historical arterial line data for neonates and older children. The pulse waveform collected with the capacitive sensing technology showed strong correlation with blood pressure values obtained through the arterial catheter. It was thus possible to use an unanchored model (without using initial measurements from other tools) to directly predict the blood pressure for this population. However, the current unanchored model predictions are less accurate than those from anchored models and do not meet the guidelines for standard deviation for all three BP parameters, SBP, DBP, and MAP, due to the limited clinical data. To fully meet the guidelines for accuracy for all three BP parameters, a model was developed to predict the blood pressure using pulse waveforms, anchored with an initial blood pressure value, as well as the age and weight of the patient.

### Study Limitations

The studies described herein were executed to determine the feasibility of using PyrAmes’ sensor technology for several applications. Much work remains to develop products that are suitable for clinical use.

The proof-of-concept demonstration and Study 1 relied on a single oscillometric cuff device to collect data for training machine-learning algorithms to extract blood pressure values from the pulse waveform shape. It had not been specifically calibrated against a known standard, and it is possible that the blood pressure reference data were systematically biased by the error of this device. While this approach was sufficient for showing feasibility of the method, significantly more data which has been carefully calibrated will be required to train generalizable algorithms suitable for clinical use.

Study 2a was designed to meet this deficiency although it was found that the quantity and quality of data collected was still insufficient to develop an algorithm which was fully generalizable across a wide range of patient ages and conditions. Accordingly Study 2b focused on neonates, a limited patient population with substantial homogeneity. Algorithms were obtained which could meet accuracy targets with the use of an initial blood pressure value, but the data set was still too small to obtain an algorithm that fully met accuracy targets without a loose anchor to an external calibration value. In general, the standard deviation without calibration was higher than the guidelines issued by the FDA while the mean average error was within target. Based on the results to date, it is believed that with additional data, it will be possible to develop models to further reduce the spread of the derived results and do not require external calibration.

## 5. Future Work

With the collection of additional clinical data across a broader range of subjects, blood pressure values, and medical conditions, it is likely that the accuracy of an unanchored model which does not require external calibration can be further improved. Moreover, collection of longitudinal data for each individual will establish the connection between medical conditions and treatments and blood pressure values, which will ensure the temporal variation of blood pressure can be tracked accurately for a given individual. To further improve temporal monitoring, mechanisms and algorithms to improve data quality and mitigate motion artifacts will need to be developed, particularly for ambulatory applications.

For accurately predicting the blood pressure across multiple individuals, it is necessary to have a large sensor waveform database covering a diversified population. A diversified population, for example, can include a broader range of race and ethnicity and income across multiple sites critical for reducing medical artificial intelligence (AI) bias. Assessing the performance of the algorithms on different demographics across locations can reduce the risk of overfitting and assess disease prevalence [104]. Blood pressure monitoring can be deployed both at hospitals and communities to raise the representativeness of the populations sampled and thereby its accuracy. The PyrAmes sensor has already conducted feasibility studies of different population groups ranging from babies to seniors, healthy to critically ill patients. Inclusion of different races and ethnicities and varying income levels will further provide robust clinical outcomes valuable for widespread uses of blood pressure monitoring.

The widespread use of wearable blood pressure sensors in the near future can be a significant stride toward improving public health. Knowing one’s blood pressure easily and frequently is one of the best ways to prevent a multitude of major illnesses including heart attack, strokes, cerebral hemorrhage, cognitive impairment and risk factors associated with hypertension. The latest progress in this field, therefore, is not only important to advance science but also to encourage preventive healthcare for a larger number of people.

## Figures and Tables

**Table 1:** Comparison of non-invasive blood pressure monitoring technologies.

	Method	Measurement	Advantages	Disadvantages	Example Companies w/FDA/EU Clearance	Example Companies w/Development Programs
Cuff	Finger, tabletop (volume clamp technology)	Continuous	Validated for adults, can be self-calibrated	Restricted mobility, bulky, expensive (>$15 K)	BMEYE, Finapres, ADI, Biopac, Edwards, CNAP	
	Finger, wearable (volume clamp technology)	Continuous	Validated for adults, can be self-calibrated	Expensive ($5 K), uncomfortable, restricts movement of hand, high power	Caretaker	
	Wrist	Intermittent	Validated for adults; established technology	Concerns about accuracy, bulky, high power	Omron, H2Care	
Cuffless	PPG	Continuous	Wearable technology widely used for heart rate; low cost; can pick up signal almost anywhere on body	Noise from ambient light, skin color; uncomfortable; high power; periodic calibration	Aktiia, BioBeat, Aura	Apple, ASUS, Samsung, Sensifree,
	PWV, PTT	Continuous	Widely studied technique; can use many combinations of sensor technology	Requires multiple sensors to determine pulse wave velocity (PWV) and pulse transit time (PTT), large training dataset, high power; many of the same issues as PPG	Sotera, Somnometics	Vital Insite, Quanttus, Scanadu, Blumio, Sibel
	Tonometer	Continuous	Established technique; validated for adults	Requires calibration, expensive, uncomfortable, restricts movement	Tensys, HealthStat	
	Capacitance	Continuous	Demonstrated for neonates; highly detailed pulse waveforms; requires minimal contact with skin for less irritation; lower power	New technology		PyrAmes, Vena Vitals

Abbreviations: **FDA**–US Food and Drug Administration; **EU**–European Union; **PWV**–Pulse wave velocity; **PTT**–Pulse transit time; **PPG**–Photoplethysmography.

**Table 2:** Feasibility Studies.

Features	Study #1: Ambulatory Adults	Study #2a: Critically Ill Adults & Children	Study #2b: NICU Infants
**Study participants**	*n* = 104, **Age** 21- > 89 y, 62% F	*n* = 124, **Age** 4–87 y, 42% F	*n* = 16, **Age** 1–8 days, 33%F **GA** 25–40 w, **Weight** 0.7–3.6 kg
**PyrAmes Data duration**	~10 min per subject (6 h total) ABPM subjects 4–24 h ea	~5 h per patient (500 h)	~10 h per patient (360 h)
**Device**	V2 Prototype (training data) Boppli Band (test data)	V3 Prototype	Boppli Band
**Sensor/Electronics size**	Area of 4-sensor array: 6 × 10 mm^2^, Electronics: 2.1 × 6.3 cm, >30 g	Area of 4-sensor array: 6 × 10 mm^2^, Electronics: 3.5 × 3.8 cm, >30 g	Area of 4-sensor array: 15 × 7 mm^2^, Electronics: 2 × 2.5 cm, 12 g
**PyrAmes device location**	Wrist	Wrist and foot	
**Source of training data**	3–100 cuff msmts per patient.	Stanford invasive arterial line (IAL) data (*n* = 899, **age** 1 day ~53 year, **Weight** 0.83 Kg~114.8 kg, 45% F), Children’s National Hospital IAL data (*n* = 40, **age** 1–2 day, **Weight** 0.5 kg~4.7 kg, 40% **F**, **GA** 23–40 wk) (~9000 h total)	
**Outcome: Meets FDA accuracy criteria?** [84] MAE (sd) < ±5 (8) mmHg	SBP: −0.3 (5.7) mmHg	SBP: −3 to 3 (>12) mmHg (no successful models)	SBP: 2.8 (5.5) mmHg DBP: −0.2 (4.6) mmHg MAP: 0.1 (4.1) mmHg
**Clinical collaborators**	Drs. Vivek Bhalla, Tara Chang, Sandra Tsai	Drs. Anita Honkanen, Chandra Ramamoorthy, Archana Varma, Alexandria Joseph	Drs. William Rhine, Anoop Rao
**Stanford study site **	Cardiology & Hypertensive clinics	Intensive Care Unit (ICU), Pediatric ICU (PICU), Cardiovascular ICU (CVICU)	Neonatal (ICU), CVICU

**Abbreviations**: **M**–Male; **F**–Female; **GA**–Gestational age; **msmts**–BP measurements; **ABPM**–Ambulatory Blood Pressure Monitor; **ANN**–Artificial neural network; **ICU**–Intensive Care Unit; **PICU**–Pediatric ICU; CVICU–Cardiovascular ICU; **NICU**–Neonatal ICU; **SBP**, **DBP** and **MAP**–Systolic, Diastolic and Mean arterial pressure; **IAL**–invasive arterial line.

**Table 3:** Results of neonate BP-model-3 (N = 16).

MAP		SBP		DBP		FDA Guidelines [84]	
MAE	sd	MAE	sd	MAE	sd	MAE	sd
0.1	4.1	2.8	5.5	−0.2	4.6	<±5	<8 mmHg

## Data Availability

Data presented in this document that were generated solely by PyrAmes are available from the corresponding author upon reasonable request. The data are not publicly available due to sensitive personal data that were collected during clinical studies on patients. Data generated or provided by Stanford University and Children’s National Hospital are restricted by the terms of contractual agreements and are available upon reasonable request with the permission of those organizations and if the requestor agrees to comply with the European General Data Protection Regulation and not to attempt to trace the origin of the data.
